# Innovations in Psychiatry: Ambulatory services for the mentally ill

**DOI:** 10.4103/0019-5545.55081

**Published:** 2009

**Authors:** H. Chandrashekar, N. R. Prashanth, C. Naveenkumar, P. Kasthuri

**Affiliations:** Department of Psychiatry, Bangalore Medical College and Research Institute, Bangalore, India

In India, mental morbidity is set to overtake that of cardiovascular diseases by 2010. There are over two crore persons with serious mental disorders and about five crore people with common mental disorders. About 30 to 35 lakh need hospitalization at any time. But, a huge treatment gap exists, with around 50 to 90% of the people not being able to access the required services.[1] Thus, there exists an urgent need to augment health care infrastructure. Moreover, the focus of care is being shifted towards community participation. In view of the above, short duration of hospitalization have become a norm. This has led to a revolving door policy where in patients get admitted at the time of crisis and discharged soon after the crisis gets resolved.

Psychiatric patients pose distinct challenges to the treating physicians, families and society at large. Aggression, agitation, confusion, violence, suicidality, non-adherence and exacerbation of psychotic symptoms are situations during which interventions are most required. Many a times, patients refuse care due to lack of insight. Family members by themselves find it impossible to convince patients to come to the hospital. Lack of knowledge among general practitioners compounds the whole problem. The consequences of such events may be dangerous and disastrous to both parties. During those times, families have expressed need for psychiatric ambulance services (in which members of the treating team could go to their homes and help them manage such patients). Existing services have been unable to handle psychiatric crisis. There have been instances where such services have simply refused to serve psychiatric patients. Also, these have been misused causing medico-legal problems as well as violation of human rights.[2] Research in the west notes that an important proportion of patients brought in by an ambulance can be described as high risk referrals.[3] Such models have included a psychiatrist accompanying the treating team.[4] Indeed, families have expressed high levels of satisfaction after interventions.[5] One similar service (in our country) to homeless mentally ill has proved successful.[6] Potential advantages of such services could be many: Improved treatment access, the capability to avert a crisis or decrease its severity, reduction in family burden, reduced criminalization of the mentally ill and cost-effectiveness. However due to gross deficiency of trained mental health professionals, inclusion of psychiatrists and qualified psychiatric nurses in the ambulance services is impractical.

In this context, ‘Ambulatory services for the mentally ill’ is an initiative taken by the Karnataka State Mental Health Authority (KSMHA), in coordination with Government of Karnataka, Rotary Club, Bangalore West, and ACMI, a non-governmental organization (NGO). The request for such services had come from NGOs formed by families of the mentally ill. Guidelines were framed and approved by the KSMHA. Funding for this project has come from NGOs and the KSMHA. This has been functioning from October 2008 onwards.

The logistics of this service consists of an ambulance van with equipments for first aid, two pilots and three trained nurses. These personnel were trained in handling psychiatric emergencies.

The ambulance staff have been provided with a cell phone. This number was advertised in mass media. After getting the call, the staff will collect background information about the patient and the caller. Then the ambulance reaches patients' residence. A written request is obtained from the family members before any intervention is made. The ambulance staff will assess the clinical situation and will assist the family to shift the patient to the hospital of their choice. It is mandatory for the family members to accompany the patient during shifting. If patient is uncooperative (usual scenario), the ambulance staff tries to counsel the patient to present himself/herself for medical evaluation. If the counseling fails, the staff suggests the family to seek admission in the hospital under various provisions of the Mental Health Act 1987. If treating physician is available, the ambulance staff under his/her supervision may administer medications. The emergency team is not authorized to use any form of physical restraint on the patient. However, if the family wishes to physically restrain the patient, safe means will be suggested. This service is free of cost and exclusive for psychiatric emergencies and operated within a specified geographic boundary.

The service is being monitored on a daily basis by the faculty of department of Psychiatry, Bangalore Medical College and Research Institute, Bangalore. Periodic review is being done by the KSMHA.

To date, sixty-nine patients have utilized the service. Fifteen were shifted with restraint, seven were administered parenteral medication. Our experience with this service has been enriching. The service has been able to avoid many crises and violent situations during shifting. Many patients who would otherwise be noncompliant to the family have been cooperative to the staff. Generally, families have been thankful. Though no empirical research has been done about the utility of this service, the fact that it has helped many needy families is irrefutable.

Ignorance and ambivalence of families, aggression towards ambulance staff, ignorance of police/judiciary towards legal provisions, problem in continuous funding for the programme are some of the difficulties faced by the ambulance service.

In conclusion, separate ambulance services for psychiatric patients is a need. The experience of running such a service is gratifying, though there are many legal, financial hurdles. Standard operating procedures need to be worked out. Empirical research is needed to plan best way to help families handling psychiatric emergencies.

## References

[1] Nagaraja D, Murthy P (2008). Mental Health Care and Human Rights.

[2] (2008). Torture at the rehabilitation centre. Deccan Herald.

[3] Spooren D, Buylaert W, Jannes C, Henderick H, Van Heeringen C (1996). Patients with psychiatric emergencies transported by an ambulance in an urban region. Eur J Emerg Med.

[4] Nordentoft M, Bjarking L, Hemmingsen R (2002). Psychiatric emergency outreach: A report on the first 2 years of functioning in Copenhagen. Nord J Psychiatry.

[5] Ampelas JF, Robin M, Caria A, Basbous I, Rakowski F, Mallat V (2005). Patient and their relatives' satisfaction regarding a home based crisis intervention provided by a mobile crisis team. Encephale.

[6] (2008). ‘Dial 100’- A model of public-private partnership. Report of the National Workshop.

